# Changes in Maxillary Sinus Structure Due to Tooth Loss and the Effects of Sex and Aging on CBCT Before Maxillary Sinus Augmentation: A Cross-Sectional Study of 120 Patients

**DOI:** 10.3390/bioengineering12030240

**Published:** 2025-02-26

**Authors:** Takumi Itokawa, Kikue Yamaguchi, Kotaro Yagi, Kazuyuki Araki, Daisuke Sato, Motohiro Munakata

**Affiliations:** 1 Department of Implant Dentistry, Showa University Graduate School of Dentistry, 2-1-1 Kita-senzoku, Ota-ku, Tokyo 145-8515, Japan; gd21-t005@grad.showa-u.ac.jp; 2 Department of Implant Dentistry, Showa University School of Dentistry, 2-1-1 Kita-senzoku, Ota-ku, Tokyo 145-8515, Japan; 120501ds@dent.showa-u.ac.jp (K.Y.); dsato.imp@dent.showa-u.ac.jp (D.S.); munakata@dent.showa-u.ac.jp (M.M.); 3 Division of Radiology, Department of Oral Diagnostic Sciences, Showa University School of Dentistry, 2-1-1 Kita-senzoku, Ota-ku, Tokyo 145-8515, Japan; araki@dent.showa-u.ac.jp

**Keywords:** aging, sex, anatomical variation, dental implant, maxillary sinus, maxillary sinus augmentation, maxillary sinus volume, missing tooth

## Abstract

Prosthesis for missing maxillary molars with dental implants often requires maxillary sinus augmentation owing to insufficient alveolar bone height. However, the anatomical structure of the maxillary sinus is a significant risk factor. This study used preoperative cone-beam computed tomography (CBCT) to investigate changes in the anatomical structure of the maxillary sinus due to tooth loss and conducted an epidemiological survey of Japanese people to determine the effects of gender and age on these changes. Preoperative CBCT scans were analyzed in patients aged ≥ 50 years with free-end maxillary molar edentulism involving ≥ 2 missing teeth seeking implant treatment. Statistical analyses were performed. One hundred and twenty participants (46 males, 74 females; mean age, 62.1 ± 7.4 years) with 3.0 ± 0.9 missing teeth and an existing bone volume of 6.2 ± 3.1 mm were included. Lateral wall thickness, sinus angle, sinus membrane thickness, maxillary sinus length and width, and the presence of the sinus septa significantly differed between missing and non-missing sides. Maxillary sinus height and sinus membrane thickness on missing side and maxillary sinus height and width on non-missing side varied significantly. Aging was associated with maxillary sinus length and width changes on the non-missing side in males, whereas no age-associated effects were identified in females. To ensure the safe execution of maxillary sinus augmentation, a thorough understanding of maxillary sinus anatomy is crucial prior to surgery.

## 1. Introduction

Prosthetic treatment involving dental implants, having gained recognition as a highly predictable approach with favorable long-term outcomes, is commonly performed to achieve both esthetic and functional restoration. However, implant placement in the maxillary molar regions frequently necessitates maxillary sinus augmentation owing to inadequate alveolar bone height, often resulting from alveolar bone resorption following tooth loss and alterations in the maxillary sinus. Anatomically, the maxillary sinus is a pyramidal cavity within the skull, with its base situated on the alveolar lateral wall and its apex extending to the zygomatic process. A close relationship exists between the maxillary sinus floor, teeth, and the alveolar bone, with changes in the sinus following tooth loss being influenced by factors such as the cause of tooth extraction, the extent of molar root protrusion into the sinus, effects of aging, and bite force [1,2,3,4,5,6,7,8,9,10,11].

Since the 2000s, clinical studies on maxillary sinus augmentation have explored various techniques and bone graft materials, establishing the procedure as a reliable and predictable treatment option for maxillary molars. In 2018, a systematic review reported a high implant survival rate of 92–100% over observation periods exceeding 5 years, regardless of the tooth loss type, surgical technique, or bone graft material used [12]. Similarly, in 2019, a systematic review and meta-analysis demonstrated a 5-year implant survival rate of 97.8%, further solidifying maxillary sinus augmentation as a well-established surgical approach [13].

Numerous reports have highlighted intraoperative and postoperative complications associated with maxillary sinus augmentation, including sinus membrane perforation, arterial injury, postoperative sinusitis, graft leakage, and wound dehiscence [14,15,16]. Risk factors contributing to these complications include anatomical features such as the ostiomeatal complex (OMC), including deviations such as septal deviation; sinus membrane thickness; the presence, orientation, and height of the septa; lateral wall thickness; sinus angle and width; palatal–nasal recess (PNR) angle; the position of the zygomaticomaxillary buttress (ZMB); and the trajectory of the posterior superior alveolar artery. Testori et al. [14] further emphasized the importance of these anatomical variations by classifying them as factors affecting the complexity of maxillary sinus augmentation. The understanding of the anatomical structure of the maxillary sinus has a significant impact on the diagnosis, treatment planning, and treatment methods for maxillary sinus augmentation. Despite this, detailed research on the effects of tooth loss in the same patient, as well as the influence of sex differences and aging on maxillary sinus morphology, is limited.

Therefore, the aim of this study was to investigate the relationship between tooth loss and maxillary sinus anatomy in the same patient, as well as the effects of sex and age, using cone-beam computed tomography (CBCT) to examine the epidemiological data in three dimensions for Japanese patients.

## 2. Subjects and Methods

### 2.1. Study Design

This cross-sectional study used CBCT images and included patients who underwent maxillary sinus augmentation for unilateral maxillary free-end loss at the Implant Dentistry Department of Showa University Dental Hospital between April 2020 and March 2023.

The inclusion criteria were as follows: (1) patients with unilateral maxillary free-end edentulism (missing two or more consecutive teeth), (2) patients over 50 years of age, and (3) patients who had undergone preoperative CBCT imaging at least 3 months after tooth extraction [4,7]. The exclusion criteria included the following: (1) patients with an existing bone volume of 10 mm or more in the missing area, (2) patients with missing maxillary anterior teeth, (3) patients with a maxillary sinus floor mucosa thickness of 5 mm or more in preoperative CBCT images, (4) patients with opaque findings such as mucous cysts or polyps in the maxillary sinus, (5) patients with paranasal sinus disease, (6) patients with respiratory conditions such as bronchitis or bronchial asthma, and (7) smokers (10 or more cigarettes per day).

This study was approved by the Institutional Review Board of Showa University Dental Hospital (approval number: SUDH0043; date: 25 August 2020).

### 2.2. Measurement Method Using CBCT Images

The images were acquired using a dental CBCT (KaVo 3D Exam; KaVo Dental Systems, Biberach, Germany). The imaging parameters were set to 120 kVp, 5 mA, an acquisition time of 8.9 s, an axial slice thickness of 0.25 mm, and an isotropic voxel size of a 16 × 16 cm image area. All the images were recorded in Digital Imaging and Communications in Medicine (DICOM) format, and the DICOM data were analyzed using simulation software (Invivo5: Anatomage, Santa Clara, CA, USA).

### 2.3. Measurement Items

#### 2.3.1. Linear Measurements of Maxillary Sinus Height

**Figure 1:**
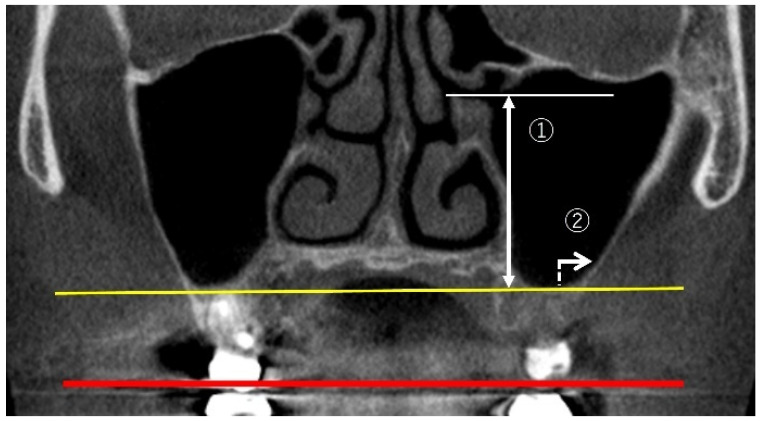
A coronal view. ① OH (sinus height): the distance from the lower edge of the ostium to the lowest point of the maxillary sinus floor (yellow line). ② LWT was measured 2 mm above the deepest point of the maxillary sinus floor (yellow line). All the lines are parallel to the occlusal plane (red line).

Measuring maxillary sinus height involved measurements of ostium height (OH) and lateral wall thickness (LWT) as illustrated in Figure 1.

OH: following the method of Benjaphalakron et al. [17], the distance from the lower edge of the ostium to the lowest point of the maxillary sinus floor was measured perpendicularly to the virtual occlusal plane in the coronal view of the CBCT image. LWT: using the same technique, the distance was measured 2 mm above the lowest point of the maxillary sinus floor.

#### 2.3.2. Measurement of PNR Angle and Maxillary Sinus Angle (MSA)

**Figure 2:**
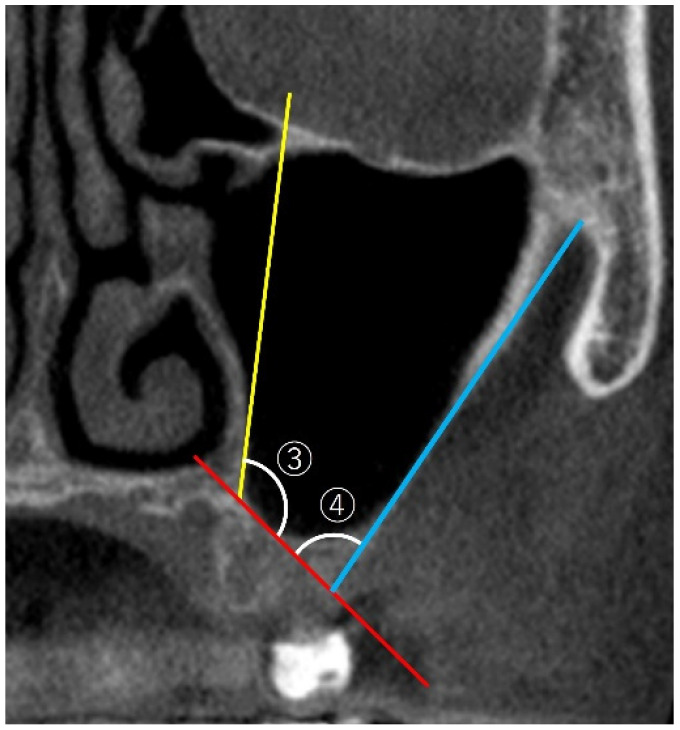
A coronal view of the cone-beam computed tomographic image. ③ PNR angle: the angle formed by two imaginary lines—one along the lower part of the lateral nasal wall (yellow line) and the other along the palatal wall of the maxillary sinus (red line)—was measured at the level of the first molar. ④ MSA: the angle formed by two imaginary lines—one along the lateral wall (blue line) and the other along the medial wall (red line) of the maxillary sinus—was measured at the level of the maxillary first molar.

The methods of PNR angle and MSA measurements are illustrated in Figure 2.

PNR: following the method of Chan et al. [18], the angle between the lower part of the lateral nasal wall and palatal wall in the maxillary sinus was measured in the coronal view of the CBCT image at the level of the first molar. MSA: the angle between the lateral and medial walls of the maxillary sinus was measured.

#### 2.3.3. Measurement of Sinus Membrane Thickness (SMT)

**Figure 3:**
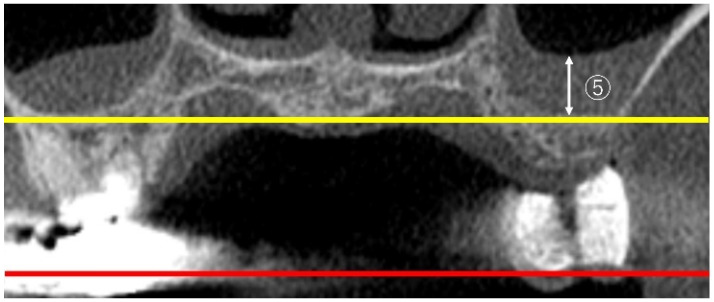
⑤ SMT: in the coronal view, the thickness of the sinus mucosa was measured perpendicularly to the deepest part of the maxillary sinus floor (yellow line). The yellow line is parallel to the occlusal plane (red line).

Following the method of Benjaphalakron et al. [17], SMT was measured perpendicularly to the deepest point of the maxillary sinus floor, as depicted in Figure 3.

#### 2.3.4. Linear Measurements of Maxillary Sinus Length

**Figure 4:**
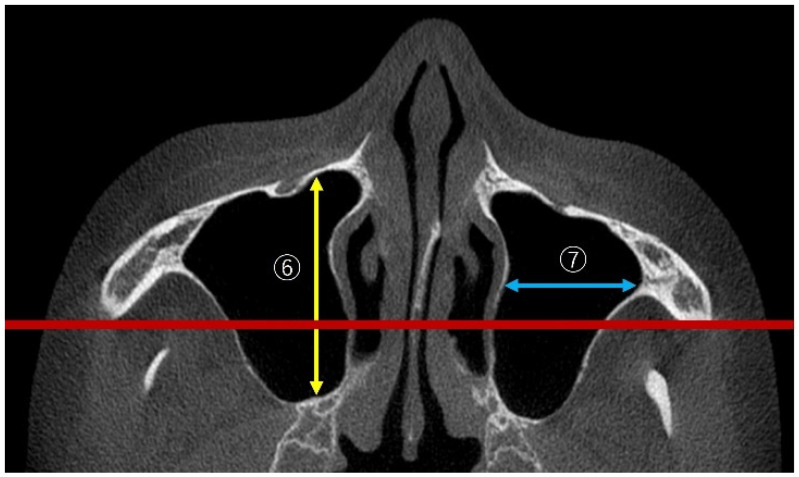
⑥ Measurements of maxillary sinus length (AP, yellow line) and ⑦ sinus width (ML, blue line): in the axial view, measurements were taken vertically to the left and right zygoma levels (red line) to determine the maximum value for AP and horizontally to determine the maximum value for ML.

The maxillary sinus length measurement involved the anteroposterior dimension (AP) and sinus width (mediolateral dimension; ML) as depicted in Figure 4. Following the method of Hettiarachchi et al. [19], the maximum values measured vertically to the zygoma level on both sides in the axial view were defined as AP, while the maximum values measured horizontally were defined as ML.

#### 2.3.5. Maxillary Sinus Septa

**Figure 5:**
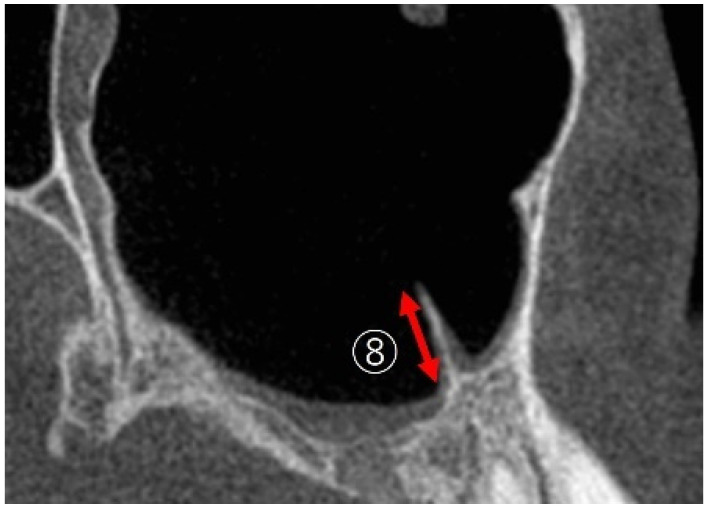
⑧ The measurement of the maximal sinus septa: the height of the septa was measured in the sagittal view.

The septa was measured following the method of Benjaphalakron et al. [17] as depicted in Figure 5. Septas with a height of 2 mm or more in the sagittal view were defined as present.

### 2.4. Statistical Analysis

Statistical analyses were conducted using the Wilcoxon *t*-test/χ^2^ square test for comparison between patients, the Mann–Whitney U-test/χ^2^ square test for sex differences, and Spearman’s correlation for associations with aging, with the significance level set at *p* = 0.05.

## 3. Results

This study included 120 patients (46 males, 74 females), with a total of 240 sinuses. The average age was 62.1 ± 7.4 years (males: 61.7 years; females: 61.4 years). The average number of missing teeth was 3.0 ± 0.9, and the average existing bone volume in the missing area was 6.2 ± 3.1 mm.

### 3.1. Effects of Tooth Loss on the Maxillary Sinus (Table 1)

#### 3.1.1. OH (Sinus Height)

In all the patients, no significant difference was observed between the missing and non-missing sides (*p* = 0.25). However, when comparing males and females, the missing side showed significantly lower values (*p* = 0.046).

**Table 1:** Effects of tooth loss.

	Missing Teeth	Non-Missing Teeth	*p*-Value
OH (mm)			
Total	33.2 ± 4.5	33.4 ± 5.1	0.25
Male	34.3 ± 7.1	35.1 ± 8.1	0.046 *
Female	32.7 ± 5.8	32.7 ± 5.4	0.43
LWT (mm)			
Total	1.90 ± 0.9	2.40 ± 1.0	2.67 × 10^−7^ **
Male	1.87 ± 1.1	2.36 ± 1.3	0.00042 **
Female	1.93 ± 1.2	2.42 ± 1.3	7.12 × 10^−5^ **
PNR (°)			
Total	120.5 ± 19.4	119.0 ± 17.9	0.20
Male	120.0 ± 28.2	117.3 ± 32.8	0.26
Female	120.8 ± 24.2	120.0 ± 23.3	0.29
MSA (°)			
Total	102.2 ± 25.4	88.7 ± 15.5	0.00025 **
Male	104.0 ± 42.7	88.5 ± 21.2	0.014 *
Female	101.1 ± 31.7	88.7 ± 21.4	0.0035 **
SMT (mm)			
Total	0.78 ± 1.3	0.37 ± 0.8	0.009 **
Male	1.01 ± 2.4	0.46 ± 1.8	0.017 *
Female	0.49 ± 1.4	0.30 ± 1.3	0.17
AP (mm)			
Total	34.9 ± 4.5	36.5 ± 3.7	1.95 × 10^−5^ **
Male	35.4 ± 7.9	36.9 ± 6.4	0.0078 *
Female	34.5 ± 4.6	34.5 ± 5.3	0.00044 **
ML (mm)			
Total	25.2 ± 4.2	26.9 ± 3.7	1.78 × 10^−7^ **
Male	25.2 ± 7.7	27.5 ± 6.7	3.14 × 10^−6^ **
Female	25.3 ± 4.9	26.6 ± 4.2	0.00021 **
Septa % (n)			
Total	43.3 (51/120)	25.8 (30/120)	0.0043 **
Male	19.6 (16/46)	17.3 (8/46)	0.039 *
Female	48.6 (36/74)	18.9 (14/74)	0.042 *

AP, anteroposterior dimension; LWT, lateral wall thickness; ML, mediolateral dimension; MSA, maxillary sinus angle; OH, ostium height; PNR, palatal–nasal recess; SMT, sinus membrane thickness; Wilcoxson *t*-test/χ square test: * *p* < 0.05; ** *p* < 0.005.

#### 3.1.2. LWT

Overall, the thickness was significantly thinner on the missing side (1.90 ± 0.9 mm) compared to the non-missing side (2.40 ± 1.0 mm) (*p* = 2.67 × 10^−7^ < 0.05). In males, the thickness was 1.87 ± 1.1 mm on the missing side and 2.36 ± 1.3 mm on the non-missing side, whereas in females, it was 1.93 ± 1.2 mm on the missing side and 2.42 ± 1.3 mm on the non-missing side. In both males and females, the missing side was significantly thinner (*p* = 0.00042 < 0.005 and *p* = 0.0000712 < 0.005, respectively).

#### 3.1.3. PNR

No difference in PNR was found between the missing and non-missing sides when comparing all patients and by sex (*p* = 0.20, *p* = 0.26, and *p* = 0.29, respectively).

#### 3.1.4. MSA

Overall, the missing side had an angle of 102.2 ± 25.4° and the non-missing side had an angle of 88.7 ± 15.5°, with the missing side being significantly larger (*p* = 2.48 × 10^−4^ < 0.005). In males, the missing side had an angle of 104.0 ± 42.7° and the non-missing side had an angle of 88.5 ± 21.2°, with the missing side being significantly larger (*p* = 0.014 < 0.05). In females, the missing side had an angle of 101.1 ± 31.7° and the non-missing side had an angle of 88.7 ± 21.4°, with the missing side being significantly larger (*p* = 0.0035 < 0.005).

#### 3.1.5. SMT

Overall, the thickness on the missing side was 0.37 ± 0.8 mm, while on the non-missing side, it was 0.78 ± 1.3 mm, with the missing side being significantly thinner (*p* = 0.009 < 0.05). In males, the thickness on the missing side was 0.46 ± 1.8 mm and on the non-missing side it was 1.01 ± 2.4 mm, with the missing side being significantly thinner (*p* = 0.017 < 0.05). However, in females, no difference was observed between the missing and non-missing sides (*p* = 0.17).

#### 3.1.6. AP and ML (Sinus Length and Width)

AP was significantly smaller on the missing side, 34.9 ± 4.5 mm, compared to 36.5 ± 3.7 mm on the non-missing side (*p* = 1.78 × 10^−7^ < 0.005), and ML was significantly smaller on the missing side, at 25.2 ± 4.2 mm, compared to 26.9 ± 3.7 mm on the non-missing side (*p* = 1.95 × 10^−5^ < 0.005). Additionally, when comparing males and females, AP in males was significantly smaller on the missing side (25.2 ± 7.7 mm) compared to the non-missing side (27.5 ± 6.7 mm; *p* = 0.00000314 < 0.005). Furthermore, in females, ML was significantly smaller on the missing side (34.5 ± 4.6 mm) than that on the non-missing side (34.5 ± 5.3 mm; *p* = 0.00044 < 0.005).

#### 3.1.7. Septa

The proportion of patients with a septa was 25.8% (30/120) on the non-missing side and 43.3% (51/120) on the missing side, with the missing side showing a significantly higher proportion (*p* = 0.0043 < 0.005). Additionally, when comparing by sex, the missing side showed a significantly higher proportion of patients in both males and females (*p* = 0.039 and *p* = 0.042, respectively).

### 3.2. Effects of Sex on the Maxillary Sinus

The effects of sex on the maxillary sinus are presented in Table 2.

#### 3.2.1. Missing Teeth

Regarding OH, the mean OH was 34.4 ± 4.8 mm in males and 32.6 ± 4.9 mm in females, with males showing significantly greater values than females (*p* = 0.029 < 0.05). However, no significant differences were observed in other measurements.

#### 3.2.2. Non-Missing Teeth

The SMT was 1.18 ± 1.6 mm in males and 0.68 ± 1.5 mm in females, with males showing significantly thicker sinus membranes (*p* = 0.045 < 0.05). The ML was 27.5 ± 4.5 mm in males and 26.6 ± 3.6 mm in females, with females showing significantly smaller values. However, no significant differences were observed in the other examined parameters.

### 3.3. Effects of Aging on the Maxillary Sinus

The effects of aging on the maxillary sinus are illustrated in Table 3.

Among factors related to aging, in males, a positive correlation was observed for PNR and a negative correlation was observed for ML on the defective side (*p* = 0.0068 and *p* = 0.0089, respectively). On the non-defective side, AP and ML showed a negative correlation (*p* = 0.049 and *p* = 0.0044, respectively). In contrast, in females, no changes related to aging were observed in any of the parameters.

## 4. Discussion

Sinus augmentation in dental implant treatment differs from other bone augmentation techniques in that multiple factors are crucial to the success of the procedure [20,21,22].

Regarding changes in maxillary sinus structure after tooth loss, it is generally believed that maxillary sinus pneumatization occurs following maxillary molar extraction [4]. Although there has been an increase in studies examining the relationship between maxillary sinus volume (MSV) and tooth loss in three dimensions using CT images, few studies have focused on these factors within the same patients, and no reports have identified which part of the volume is affected. Velasco-Torres et al. [7] compared MSV in dentate, partially edentulous, and edentulous patients and reported that the volume of partially edentulous and edentulous patients was significantly smaller than that of dentate patients. Similarly, Sharan and Madjar [2] reported that the MSV decreased as tooth loss increased, but in completely edentulous patients, the MSV increased due to pneumatization caused by bone loss and the associated decrease in bone stress. Furthermore, Yamaguchi et al. [9] measured the MSV of the defective and non-defective areas of the same patient who had undergone maxillary sinus floor augmentation surgery and reported that the MSV of the missing side was significantly smaller. Although volume measurements were not performed in this study, the AP and ML on the missing side were significantly smaller when comparing all patients and by sex, indicating that MSV was reduced due to tooth loss. The results also showed that sinus length and width had a stronger effect on volume reduction than sinus height, which is consistent with other reports.

Regarding the effects of sex and aging, Takahashi et al. [23] reported the average bilateral MSV to be 31.3 cm^3^ overall, 29.6 cm^3^ for females, and 32.9 cm^3^ for males. The bilateral MSV decreased with age, with no significant sex differences. Based on this, it was reported that the sex difference becomes smaller as the MSV decreases with age. Barros et al. [24] found that sinus width was significantly larger in males than females, but there was no significant difference between the sexes in sinus height or length. Additionally, Teixeira et al. [25], Mathew and Jacob [26], and Paknahad et al. [27] reported that sinus height was the best discriminant measurement for estimating sex. Furthermore, Belgin et al. [28] conducted a retrospective study investigating changes in MSV by age (18–55 years or more) and sex and reported no statistically significant difference between the left and right MSV, with significantly larger MSV in males than females, and that MSV varied considerably depending on age group. Velasco-Torres et al. [7] found that the MSV in patients with missing teeth was not related to sex or tooth loss condition but was affected by age, with volume decreasing with age. Yamaguchi et al. [9] also measured MSV in people in their 50 s and reported an average of 24.5 cm^3^ for males and 20.8 cm^3^ for females, with males showing significantly larger volumes, although no significant difference was observed, and volume decreased with age. The results of this study are similar to those of other studies; however, because the subjects were relatively older patients who had undergone implant treatment aged 50 years or older, it was difficult to distinguish between the effects of aging and sex differences. Nevertheless, it was revealed that the sinus height was greater in males than in females, and that MSV (especially sinus width) in males decreased with age, regardless of whether they had a missing or non-missing side.

Regarding the relationship between LWT and maxillary sinus augmentation, it has been reported that a thick LWT often complicates surgical procedures during maxillary sinus augmentation. A thicker LWT may lead to increased bleeding due to more extensive vascularization of the cancellous bone compared to the cortical bone [29,30,31,32,33]. Additionally, sinus membrane perforation, the most frequent complication of maxillary sinus augmentation, is influenced by LWT. When the LWT was 2 mm or thicker, the sinus membrane perforation rate was 56.4%, whereas when the LWT was 1 mm or thinner, it decreased to 12.1% [34]. In this study, measurements were taken at the first molar-equivalent site located anterior to the ZAC line, where bony window formation for maxillary sinus augmentation is typically performed. The results showed that the missing side was significantly thinner than the non-missing side when compared within the same patient. Regarding LWT, Monje et al. [35] reported that LWT tended to be thicker from premolars to molars, and although there was a significant difference between the sexes in the mean LWT, no correlation was found. However, there was a positive correlation with age (0.0017 mm/year). They also investigated the effect of tooth loss duration on side wall thickness and reported that the longer the tooth loss duration was, the thinner the LWT was. Furthermore, Khajehahmadi et al. [36] found no significant difference between the missing and non-missing sides, but the non-missing side tended to have a thicker side wall. The results of this study are consistent with those of Monje et al. [35] and Khajehahmadi et al. [36]. However, other factors, such as the time since tooth extraction and bone volume, have also been reported to affect LWT [37,38], indicating that further investigation is needed.

The sinus septa is a protruding bony structure in the maxillary sinus. A recent systematic review and meta-analysis [39] reported that the incidence of the septa was 33.2% at the sinus level and 41.0% at the patient level. Hungerbuhler et al. [40] explained that there are two types of sinus septas depending on their occurrence: primary septas due to congenital differences and secondary septas formed by the irregular cavitation of the maxillary sinus after maxillary tooth loss. The prevalence of the septa at the defect site was 26.6% [1,41]. Numerous papers have reported that the relationship between the septa and maxillary sinus augmentation is a risk factor for sinus membrane perforation [42,43]. Al-Dajani [44] reported in a meta-analysis that thin membranes and the sinus septa increase the risk of sinus membrane perforation. Furthermore, when a septa with a height of 2 mm or more is present in the maxillary sinus, it is more likely to cause sinus membrane perforation because of its strong adhesion to the sinus membrane, and patients with a septa have an eight-fold higher sinus membrane perforation rate than patients without a septa. The septa can be considered the most important structure that may be a risk factor for maxillary sinus augmentation [20,45]. However, since the formation of secondary septas occurred after tooth extraction in this study, it is quite possible that they may be affected by the period after tooth extraction, and this should be a topic for future research.

The structural changes in the maxillary sinus, which were the focus of this study, are influenced by OMC factors, such as a deviated nasal septum, the cause of the need for tooth extraction, the period since extraction, and the number of missing teeth. Given that the subjects were patients with implants, who were relatively older (over 50 years old), this study is limited in the sense that it did not investigate the relationship with the actual onset of complications after sinus augmentation. Therefore, further studies are needed to validate the aforementioned results, including comparisons with postoperative evaluations.

## 5. Conclusions

The results of this study revealed the following:

1. MSV, particularly sinus length and width, significantly decreased with tooth loss. Additionally, LWT decreased, MSA significantly increased, and the proportion of the sinus septa increased.
2. Regarding sex differences in the maxillary sinus, sinus height (OH) was significantly greater in males on the non-missing side, and LWT (SMT) was significantly thicker in males on the missing side.
3. Regarding the effects of aging on the maxillary sinus, in males, PNR increased and ML decreased on the missing side with age, while AP and ML decreased on the non-missing side with age. However, no effects of aging were observed in females.

To ensure the safe execution of maxillary sinus augmentation, a thorough understanding of maxillary sinus anatomy is crucial prior to surgery.

## Figures and Tables

**Table 2:** Effects of sex.

		Males	Females	*p*-Value
Missing teeth	OH (mm)	34.4 ± 4.8	32.6 ± 4.9	0.029 *
	LWT (mm)	1.87 ± 0.7	1.93 ± 1.1	0.36
	PNR (°)	120.0 ± 22.2	120.8 ± 20.8	0.47
	MSA (°)	104.0 ± 29.0	101.1 ± 27.3	0.396
	SMT (mm)	1.18 ± 1.6	0.68 ± 1.5	0.045 *
	AP (mm)	35.4 ± 5.3	34.5 ± 4.6	0.146
	ML (mm)	25.2 ± 5.2	25.3 ± 4.2	0.356
	Septa (%)	39.1	45.9	0.46
Non-missing teeth	OH (mm)	35.1 ± 5.4	32.3 ± 5.4	0.0039 **
	LWT (mm)	2.36 ± 0.9	2.42 ± 1.1	0.50
	PNR (°)	117.3 ± 19.1	120.0 ± 20.0	0.25
	MSA (°)	88.5 ± 14.4	88.7 ± 18.4	0.396
	SMT (mm)	0.68 ± 1.5	0.50 ± 1.4	0.291
	AP (mm)	37.0 ± 4.4	36.2 ± 3.9	0.162
	ML (mm)	27.5 ± 4.5	26.6 ± 3.6	0.044 *
	Septa (%)	19.6	29.7	0.31

AP, anteroposterior dimension; LWT, lateral wall thickness; ML, mediolateral dimension; MSA, maxillary sinus angle; OH, ostium height; PNR, palatal–nasal recess; SMT, sinus membrane thickness; Mann–Whitney U-test/χ square test: * *p* < 0.05; ** *p* < 0.005.

**Table 3:** Effects of aging.

		r	*p*
Missing teeth	OH		
	Male	−0.089	0.28
	Female	0.014	0.45
	LWT		
	Male	0.058	0.35
	Female	−0.074	0.26
	PNR		
	Male	0.36	0.0068 *
	Female	−0.12	0.15
	MSA		
	Male	−0.16	0.15
	Female	−0.09	0.22
	AP		
	Male	−0.066	0.48
	Female	−0.06	0.303
	ML		
	Male	−0.348	0.0089 *
	Female	0.053	0.32
Non-missing teeth	OH		
	Male	−0.13	0.19
	Female	0.037	0.38
	LWT		
	Male:	0.064	0.34
	Female	−0.014	0.45
	PNR		
	Male	0.11	0.23
	Female	−0.038	0.37
	MSA		
	Male	0.118	0.22
	Female	−0.099	0.20
	AP		
	Male	−0.243	0.049 *
	Female	0.12	0.15
	ML		
	Male	−0.38	0.0044 **
	Female	0.029	0.40

AP, anteroposterior dimension; ML, mediolateral dimension; MSA, maxillary sinus angle; OH, ostium height; LWT, lateral wall thickness, PNR, palatal–nasal recess; Spearman’s correlation: * *p* < 0.05; ** *p* < 0.005.

## Data Availability

The data that support the findings of the study are available from the corresponding author upon reasonable request.

## Abbreviations

The following abbreviations are used in this manuscript:

CBCT	Cone-beam computed tomography
AP	Anteroposterior dimension
ML	Mediolateral dimension
MSA	Maxillary sinus angle
MDPI	Multidisciplinary Digital Publishing Institute
LWT	Lateral wall thickness
OH	Ostium height
OMC	Ostiomeatal complex
PNR	Palatal–nasal recess
SMT	Sinus membrane thickness
ZMB	Zygomaticomaxillary buttress
DOAJ	Directory of Open Access Journals
TLA	Three-letter acronym
LD	Linear dichroism
